# Supplementary material for: Subjective risk and associated electrodermal activity of a self-driving car passenger in an urban shared space
Source: PLoS One. 2023 Nov 30;18(11):e0289913. doi: 10.1371/journal.pone.0289913 (PMC10688955; doi:10.1371/journal.pone.0289913)
Supplement: S1 Table — (DOCX) [file pone.0289913.s001.docx]

S1 Table – Descriptive Statistics of the Indicator Distributions.

| **Indicator** | **N** | **M** | **SD** | **Skeweness** | **Kurtosis** |
| --- | --- | --- | --- | --- | --- |
| *Initial* | | | | | |
| iSA | 864 | 2.448 | 1.987 | 1.073 | 0.970 |
| mSA | 864 | 0.487 | 0.287 | 0.147 | -0.827 |
| *Transformed* | | | | | |
| iSA^1/2^ | 864 | 0.000 | 0.985 | -0.095 | -0.462 |
| mSA^1/3^ | 864 | 0.000 | 0.985 | -0.127 | -0.504 |

Note. To better fit a Gaussian distribution in skewness and kurtosis, the indicators iSA and mSA were elevated to the 1/2 and 1/3 powers, respectively.
