# Supplementary material for: Subjective risk and associated electrodermal activity of a self-driving car passenger in an urban shared space
Source: PLoS One. 2023 Nov 30;18(11):e0289913. doi: 10.1371/journal.pone.0289913 (PMC10688955; doi:10.1371/journal.pone.0289913)
Supplement: S2 Table — (DOCX) [file pone.0289913.s002.docx]

S4 Table – Indicator Means as Function of the Seven Independent Factors.

|  | **SA indicators** | | **SCR indicators** | |
| --- | --- | --- | --- | --- |
| **Level** | **mSA** | **iSA** | **mSCR** | **nSCR** |
| *Angle* |  |  |  |  |
| Pi/6 | -0.11 | 0.14 | 0.59 | 0.82 |
| Pi/3 | 0.11 | -0.14 | 0.67 | 0.66 |
| *Margin* |  |  |  |  |
| 2.5 m | -0.41 | -0.41 | 0.61 | 0.72 |
| 1.5 m | 0.41 | 0.41 | 0.66 | 0.76 |
| *Order* |  |  |  |  |
| Second | -0.31 | -0.43 | 0.67 | 0.66 |
| First | 0.31 | 0.43 | 0.59 | 0.82 |
| *Orientation* |  |  |  |  |
| Back | -0.04 | -0.03 | 0.63 | 0.75 |
| Face | 0.04 | 0.03 | 0.63 | 0.73 |
| *p-Speed* |  |  |  |  |
| 5.5 kph | -0.09 | 0.05 | 0.57 | 0.75 |
| 7.5 kph | 0.09 | -0.05 | 0.69 | 0.73 |
| *Side* |  |  |  |  |
| Left | -0.11 | -0.08 | 0.65 | 0.75 |
| Right | 0.11 | 0.08 | 0.62 | 0.73 |
| *v-Speed* |  |  |  |  |
| 20 kph | -0.06 | -0.06 | 0.64 | 0.75 |
| 30 kph | 0.06 | 0.06 | 0.62 | 0.73 |

Note. The indicators mSA and iSA were centred. Consequently, for specific factors, the mean value of a level is the opposite of that of the other level. This is not the case for the mSCR and iSCR indicators, which were only scaled.
