# Supplementary figures and images for: Subjective risk and associated electrodermal activity of a self-driving car passenger in an urban shared space
Source: PLoS One. 2023 Nov 30;18(11):e0289913. doi: 10.1371/journal.pone.0289913 (PMC10688955; doi:10.1371/journal.pone.0289913)

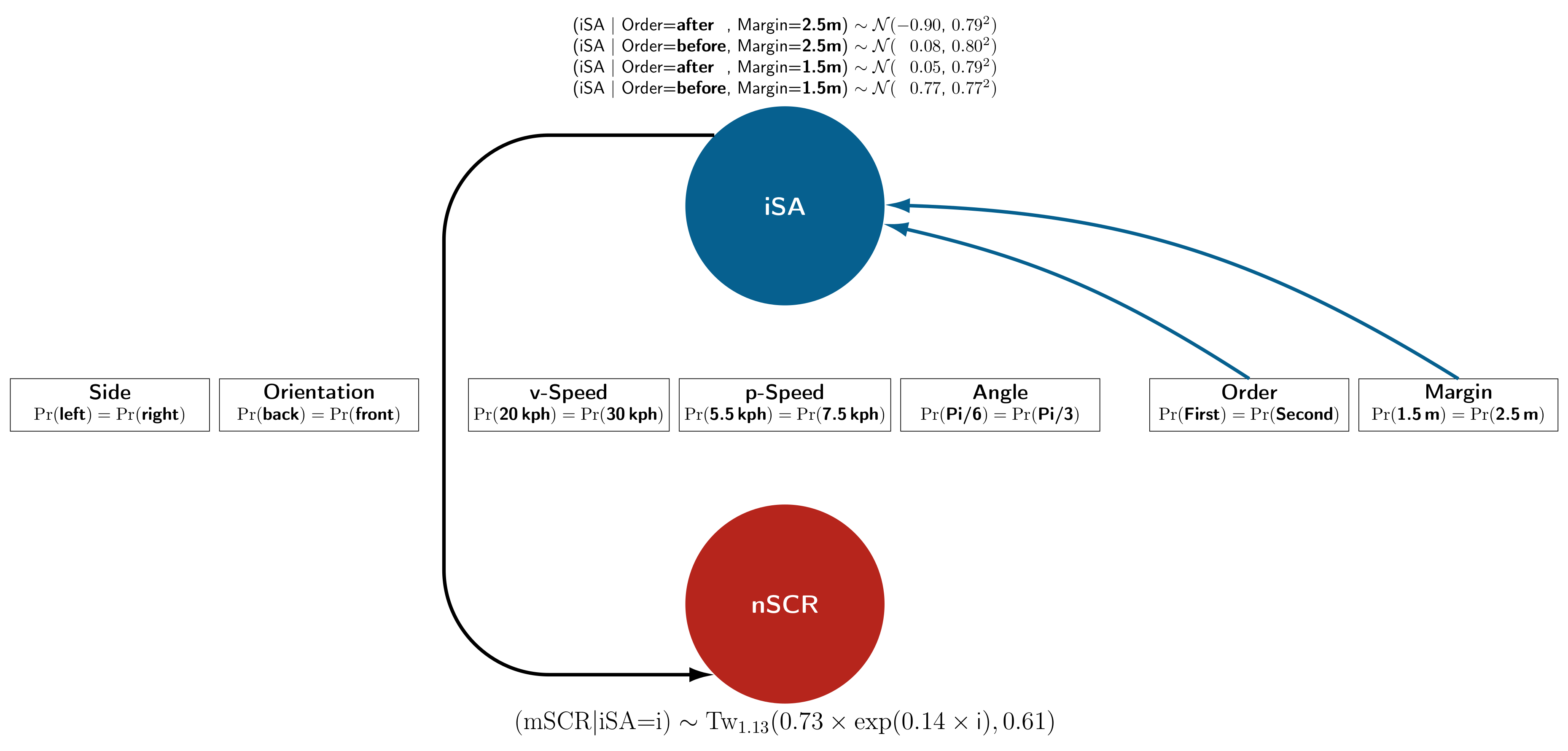


S2 Figure - Best Bayesian Network with Detailed Distributions for iSA and nSCR indicators.

Supplement: S1 Fig — (DOCX) [file pone.0289913.s003.docx]

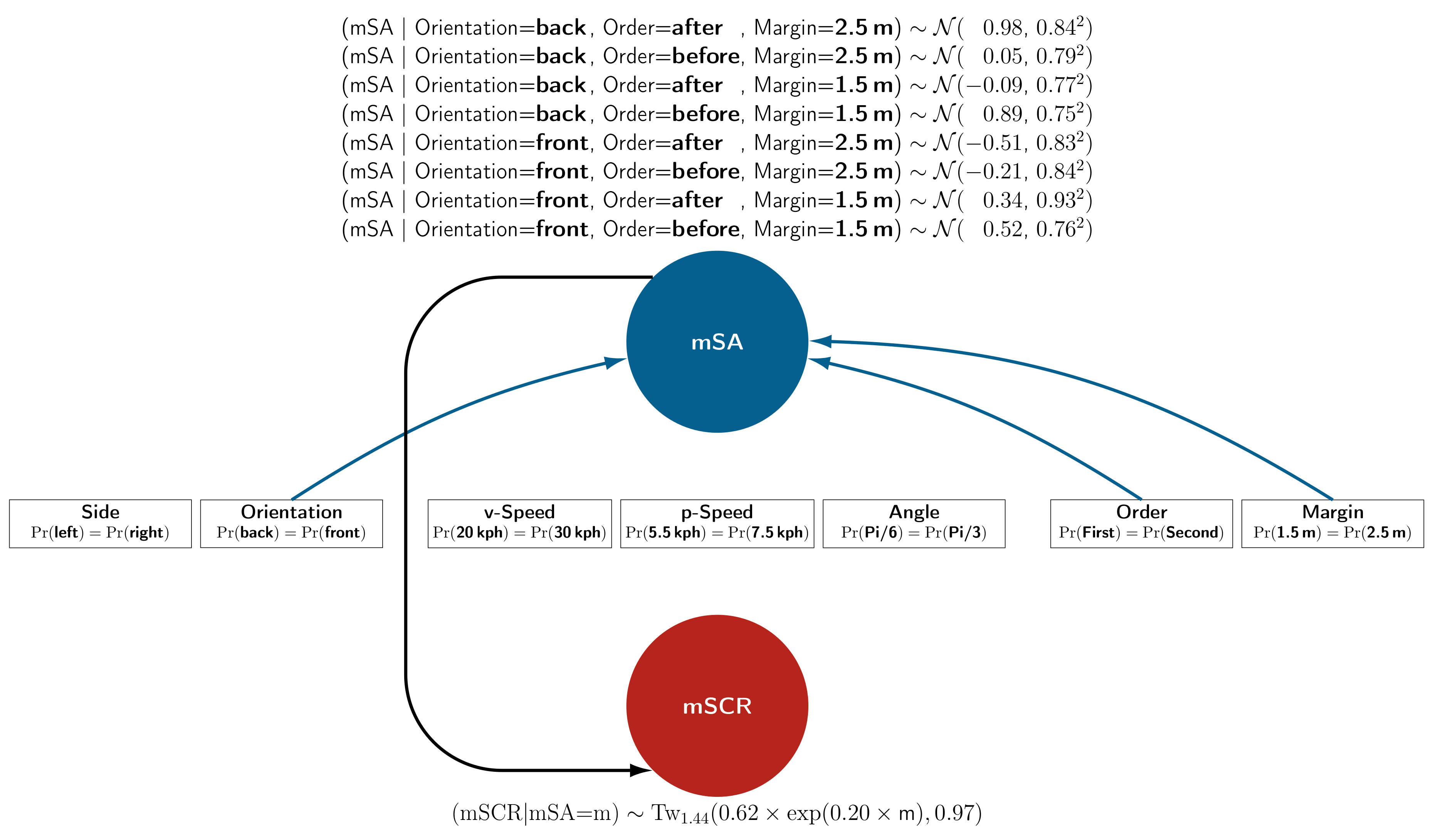


S3 Figure – Best Bayesian Network with Detailed Distributions for mSA and mSCR indicators.

Supplement: S2 Fig — (DOCX) [file pone.0289913.s004.docx]
