# Supplementary material for: Subjective risk and associated electrodermal activity of a self-driving car passenger in an urban shared space
Source: PLoS One. 2023 Nov 30;18(11):e0289913. doi: 10.1371/journal.pone.0289913 (PMC10688955; doi:10.1371/journal.pone.0289913)
Supplement: Attachment — Submitted filename: Response to reviewers.docx [file pone.0289913.s005.docx]

Dear Dr. Wollstadt

Thank you for handling this submission so efficiently. Many thanks also to the two reviewers for their comments, which we hope helped to improve the manuscript. You will find below a point-by-point response to their requests. All changes, with the exception of minor spelling and grammar corrections, are highlighted in yellow in the revised manuscript.

**Reviewer #1:**

**Summary

The paper investigates the perceived collision risk of a self-driving car’s passenger when driving in shared traffic spaces and encountering pedestrians crossing the way. Concretely, the authors evaluate the perceived risk by varying seven factors of the dynamics of the driving situation in a driving simulator. The user’s perceived risk is then measured by an electrodermal activity sensor and an analogue potentiometer allowing the user to indicate its feeling of risk. With Bayesian networks, the authors finally are able to analyze the relationship of the driving situation factors with the measured perceived risk values.

**Strengths

The paper is very well written, and the conclusions of the analysis are supported well by the data. We would like to thank the authors for their submission. The paper evaluates in detail pedestrian-vehicle interactions and helps the research community to understand the factors that influence the passenger’s feeling of an autonomous vehicle. We therefore recommend an acceptance with minor revisions.

Thank you very much for your kind words. It is much appreciated.

**Weaknesses

The authors already published similar work that shows the relationship between some driving situation factors based on Bayesian networks. The contribution is only a wider evaluation with seven factors that are analyzed. The scientific value of the paper lies only on the experimental additions.

It is true that we have implemented a similar methodology in a paper published in 2021, but this new experiment is not a simple variation. Through the number of factors manipulated, it is the complexity of the vehicle-pedestrians interaction that has considerably increased. It is also the robustness of the modelling approach that was put to the test.

A discussion on how objective risk models, such as the mentioned TTI, relate to the findings with the EDA are given. However, it would still be interesting to compare the risk value from TTI quantitatively and show how the perceived risk could improve TTI measures in pedestrian-vehicle interactions in more detail.

This is an interesting question, which we have already attempted to answer (Petit et al. 2020). It is, however, a different kind of work from that reported in this manuscript. This question has therefore been added to the new "limitations and perpectives" section (p 26, l 689-694).

Finally, we could not access the provided link with the data supporting the paper. Please provide the data for the paper.

Sorry about that. The address was missing a hyphen. This has been corrected.

**Minor revisions

1. The background and reasons for the choice of the used methods are not given in sufficient detail. It should be described or rewritten in a more understandable way why Bayesian networks were chosen for the evaluation of the relationship of the driving situation factors with the perceived risks, why the factors were treated as bi-modal random variables and why a Tweedie distribution was chosen to model the electrodermal activity responses indicators (see page 16).

In this kind of interdisciplinary work between psychology and applied maths, it's always difficult to find the right level of explanation. Based on your comments, we've tried to better formulate the justification for some choices:

- The justification of using Bayesian networks has been extended on p 15, l 377-383.
- In Bayesian networks, a probability distribution must be assigned to each variable (factor or dependent variable). In this case, the factors have been treated as bimodal random variables, since the factor modalities appear randomly from the participants' point of view. This has been specified on p 16, l 417-419.
- The paragraph presenting the tweedy distribution has been reorganized to begin with the reasons why this type of distribution is particularly well suited to the analysis of SCR indicators (p 17, l 432-434).

2. The v-speed variation is only in between the range of 20-30 kph. This could have an effect that the factor v-speed is less influencing the perceived risk value. We recommend varying the velocity between 10 – 50 kph because this is realistic for shared space scenarios or to describe this circumstance in the experiments.

Actually, speed limits in shared spaces are generally quite low. Although they may vary according to local regulations and design considerations, common speed limits in shared spaces are generally between 20 and 30 km/h). This is an essential condition for safe interactions between vehicles and pedestrians. However, you raise a valid point, that of generalizing the results to larger ranges of values than those we have manipulated. This point has been added to the discussion, p 26, l 682-688.

3. It should be discussed what the sim-to-real gap is for the experiments. For example, the modeling of the pedestrians walking, or facial features could be deviating from reality. These can change the results of the experiments.

Agreed. The limitations of driving simulator studies are now addressed on p 27, l 708-713.

4. We recommend changing the title to “Perceived Risk Assessment and Associated Electrodermal Activity of a Self-Driving Car Passenger in an Urban Shared Space” to highlight the perceived risk assessment because there are many works for objective risk assessment, such as TTI, which is not analyzed in this paper.

Agreed. The title has been changed, but rather than “Perceived Risk Assessment », we opted for « Subjective risk ».

**Other comments

1. Please rewrite the note description in Figure 3 on page 12, it is currently hard to understand.

The note has been edited. We hope it reads better now.

2. Figure 5 is referred to as Figure 5A on page 14. There is no sub-figure A.

Corrected. Thanks for the sharp eye.

3. The abbreviation BIC score is not written out when used the first time.

We modified the text so that the acronym is placed right after the full name (Bayesian information criterion)

**Reviewer #2: ## Content:**

Dependence between risk metrics:

You mention early that there are indicators for dependence between risk indicators in prior work (Petit et al. 2021). However, in the subsequent argument you mainly appear to be concerned with their complementarity, e.g., different sensitivities to low-risk situations (I assume as an argument for using both), than with possible downsides of the dependence for your investigation. When reading that part I wondered whether it might not be a methodological issue to let the active thinking about risk for the real-time subjective evaluation bias overall risk perception (and skin conductance).

On a similar note, I believe that the four crash situations may have strongly altered alertness. This might have eliminated over-trust, which may be observable within the population or specific user groups.

In the discussion a more critical examination of such introduced biases would be appreciated.

It's an interesting fundamental question, but we don't see it as a methodological issue. On the contrary, the experimental scenario was designed to be as coherent as possible. The situation in which the participants were placed corresponded to that of a passenger passing through a shared space, monitoring the situation during this time. Risk had to be continuously monitored, and the measure of perceived risk we used corresponds to this situation. It is precisely to reinforce the idea that the system is not infallible (i.e that risk exists) and that it must be supervised that we have introduced collision situations. As a result, overconfidence has been prevented. To clarify all this, several additions have been made to the research aim section (p 7, l 190-194) and in the method section (p10, l 268-269).

I do appreciate your style of data analysis and that this analysis does indeed collect some of the questions that I had when reading about the study setup.

Thank you. We are glad to have succeeded in presenting these results in a satisfactory way.

4.1

It would be great if you could cite some examples on dynamic human–machine interfaces to improve collision risk estimation that could be tested using your measures.

Proposals are now formulated p 27, l 698-702.

\## Language & style:

Introduction: Related work all presented in present tense. Past tense would often be more appropriate and has been correctly used in, e.g., Section 1.3.

The manuscript was proofread before submission by a professional editor, who suggested that we write in the present tense, but to tell the truth, it seemed a little odd. Your comment confirmed this. We therefore carried out a detailed re-reading of the whole document and converted the present tense into past tense where it seemed more natural.

Figure 3: Use larger axis labels.

Done.

Section 1.2: "Studies have proven"

Studies cannot prove but rather indicate, suggest, etc.. A proof belongs to logic, mathematics, and perhaps law.

Corrected.

2.3 "intercepted" -> "intersected"

Corrected.

Some explanations are given multiple times. Such redundancy can be reduced easily (e.g., Type 1 and type 2 process explanations).

We have not followed you on this point. A certain level of redundancy is sometimes useful, especially when it comes to key concepts with which not every reader is necessarily familiar. The reminder of what is meant by Type I and Type II processes takes just a few lines. Removing them would probably force many readers to return to the introduction.

\## PLOS requirements:

3. Download link for data given in paper but did not appear to be functional when tested (no connection to server)

Corrected
